# Poor personal protective equipment practices were associated with heat-related symptoms among Asian healthcare workers: a large-scale multi-national questionnaire survey

**DOI:** 10.1186/s12912-024-01770-x

**Published:** 2024-03-01

**Authors:** Hsiao-Yu Yang, Hsiu-Ling Chou, Clarence Hong Wei Leow, Ching-Chiu Kao, D. Daniel, Vena Jaladara, Levina Chandra Khoe, P K Latha, Yodi Mahendradhata, Phuong Minh Nguyen, Rujipas Sirijatuphat, Dewi Sumaryani Soemarko, Vidhya Venugopal, Kai Zhang, Jason Kai Wei Lee

**Affiliations:** 1 Institute of Environmental and Occupational Health Sciences, National Taiwan University College of Public Health, 10055 Taipei, Taiwan; 2 Department of Public Health, National Taiwan University College of Public Health, 10055 Taipei, Taiwan; 3 Department of Environmental and Occupational Medicine, National Taiwan University Hospital, 100225 Taipei, Taiwan; 4 Population Health Research Center, National Taiwan University, 10617 Taipei City, Taiwan; 5 Department of Nursing, Asia Eastern University of Science and Technology, 220303 New Taipei City, Taiwan; 6 Department of Nursing, Far Eastern Memorial Hospital, 220216 New Taipei City, Taiwan; 7 Human Potential Translational Research Programme, Yong Loo Lin School of Medicine, National University of Singapore, 117593 Singapore, Singapore; 8 Campus for Research Excellence and Technological Enterprise (CREATE), National University of Singapore, 138602 Singapore, Singapore; 9 Department of Nursing, Wan Fang Hospital, Taipei Medical University, 116079 Taipei, Taiwan; 10 School of Nursing, College of Nursing, Taipei Medical University, 110301 Taipei, Taiwan; 11 Department of Health Behaviour, Environment and Social Medicine, Faculty of Medicine, Public Health and Nursing, Universitas Gadjah Mada, 55281 Yogyakarta, Indonesia; 12 Department of Community Medicine, Faculty of Medicine, Universitas Indonesia, 10430 Jakarta, Indonesia; 13 Sri Ramachandra Institute of Higher Education & Research, 600116 Chennai, Tamil Nadu India; 14 Center for Tropical Medicine, Faculty of Medicine, Public Health and Nursing, Universitas Gadjah Mada, 55281 Yogyakarta, Indonesia; 15 Department of Health Policy and Management, Faculty of Medicine, Public Health and Nursing, Universitas Gadjah Mada, 55281 Yogyakarta, Indonesia; 16 Department of Military Occupational Medicine, Vietnam Military Medical University, Hanoi, Vietnam; 17 Division of Infectious Diseases and Tropical Medicine, Department of Medicine, Faculty of Medicine Siriraj Hospital, Mahidol University, 10700 Bangkok, Thailand; 18 Department of Environmental Health Sciences, School of Public Health, University at Albany, State University of New York, 12144 Rensselaer, NY USA; 19 Department of Physiology, Yong Loo Lin School of Medicine, National University of Singapore, 117593 Singapore, Singapore; 20 Heat Resilience and Performance Centre, Yong Loo Lin School of Medicine, National University of Singapore, Block MD9, 2 Medical Drive Level 4, 117593 Singapore, Singapore

**Keywords:** Heat strain, Thermoregulation, PPE, Heat management strategies, Factor analysis, Structural equation modelling

## Abstract

**Background:**

It is pertinent to understand the perceptions of healthcare workers (HCWs) with their associated personal protective equipment (PPE) usage and heat strain symptoms experienced to effectively combat the negative effects of heat stress during treatment and care activities.

**Methods:**

We evaluated the associated heat stress perceived by HCWs across Asia and validated a questionnaire on perceptions of heat stress, associated PPE usage, and heat strain symptoms experienced. The questionnaire was administered to 3,082 HCWs in six Asian regions. Factor analyses, including Cronbach’s alpha, assessed the questionnaire’s validity and reliability. Structural equation modelling analysed the effects of knowledge, attitudes and practices, and heat strain symptoms.

**Results:**

The questionnaire was found to be reliable in assessing HCWs’ knowledge, and attitudes and practices towards heat stress and PPE usage (both Cronbach’s alpha = 0.9), but not heat strain symptoms (Cronbach’s alpha = 0.6). Despite knowledge of heat stress, HCWs had negative attitudes and practices regarding PPE usage (β1 = 0.6, *p* < 0.001). Knowledge (path coefficient = 0.2, *p* < 0.001), and negative attitudes and practices (path coefficient = 0.2, *p* < 0.001) of HCWs towards heat stress and PPE usage adversely affected symptoms experienced.

**Conclusions:**

The questionnaire was not reliable in assessing symptoms. HCWs should, nevertheless, still self-assess their symptoms for early detection of heat strain. To effectively attenuate heat strain, understanding HCWs’ attitudes and practices towards PPE usage should guide policymakers in implementing targeted heat management strategies.

**Supplementary Information:**

The online version contains supplementary material available at 10.1186/s12912-024-01770-x.

## Background

From January 2020 to May 2023, the world was plagued by the coronavirus disease 2019 (COVID-19) pandemic. Since then, over 268 million cases have been recorded across the Western Pacific and Southeast Asia, with the number of deaths exceeding 1.2 million [1]. Despite wide availability of vaccinations, infections continued to rise due to the emergence of new variants and outbreak clusters. To combat the pandemic, a resilient healthcare system was necessary.

Dealing with infection control and heat stress exposure simultaneously was a new challenge for healthcare workers (HCWs) [2]. HCWs worked longer hours and more shifts due to high patient count and manpower shortages [3, 4]. Additionally, HCWs were required to wear personal protective equipment (PPE) when treating patients to reduce risk of viral transmission. PPE usage prevents heat dissipation via evaporative cooling and heat exchange with the environment, which can increase heat strain [5–7]. Compounded by rising global temperatures due to climate change [8], the heat stress experienced by HCWs is worsened by environmental stressors when working in warm outdoor conditions [9, 10]. Beyond their workplace, increasing temperatures can also affect HCWs, such as during night sleep, by further exposing them to dehydrating conditions [4]. With the known negative effects of prolonged heat stress exposure on HCWs’ health and well-being, and also knock-on effects on the patients under their dedicated care [3, 7], it is imperative to understand and alleviate the heat stress experienced by HCWs.

Understanding HCWs’ perceptions of heat stress, associated PPE usage, and heat strain symptoms experienced is an important step in developing targeted management strategies. Surveys have been used to understand the knowledge, and attitudes and practices of HCWs who wear PPE during the pandemic [3, 4]. However, the overall validity of such self-administered questionnaires remains unknown. It is important for questionnaires to be validated so their results can be appropriately interpreted [11]. This may be achieved using factor analysis, which studies the relationships between constructs within the questionnaire items to determine how close responses on different constructs relate to one another [11]. Exploratory factor analysis identifies questionnaire items from the same construct, and removes those that do not belong [11]. Subsequently, confirmatory factor analysis specifies the relationship among the confirmed constructs and questionnaire items [11, 12]. Through this approach, policymakers will be able to develop more effective solutions which target the root cause of the perceived heat stress by HCWs when working in PPE.

We aimed (i) to evaluate the heat stress perceived by HCWs engaged in different activities across Asia and (ii) to use factor analysis to validate a previously administered questionnaire on their perceptions of heat stress, associated PPE usage, and heat strain symptoms experienced [3].

## Methods

A questionnaire survey was carried out in six Southeast and South Asian countries and regions, namely India, Indonesia, Singapore, Taiwan, Thailand, and Vietnam, from May 2020 to July 2022. HCWs were invited to participate anonymously in the questionnaire either physically, or via an online platform in regions with larger land area and less-accessible hospitals where physical questionnaires may not be feasible. This study received ethical approval from the Research Ethics Committee of National Taiwan University (Taiwan; 202106HM031), Sri Ramachandra Institution of Higher Education and Research (India; IEC-NI/17/APR/59/54), National Healthcare Group Domain Specific Review Board (Singapore; 2020/00590), Siriraj Institutional Review Board (Thailand; Si277/2020), Vietnam Military Medical University Ethics Committee for Biomedical Research (Vietnam; 4812/QD-HVQY), Medical and Health Research Ethics Committee (Indonesia; KE/FK/0302/EC/2021) and Universitas Indonesia (Indonesia; KET-566/UN2.F1/ETIK/PPM.00.02/2021). All methods were performed in accordance with the relevant guidelines and regulations. The respondents provided informed consent before commencing the questionnaire. For physical questionnaires, written consent was obtained. For online questionnaires, consent was obtained electronically before participants could fill the questionnaire. To avoid duplicates, participants were asked to fill the questionnaire only once. An English version of the questionnaire is presented as Supplementary File 1.

### Questionnaire

The questionnaire was initially developed by the authors in a previously published study to assess HCWs’ perceptions of heat stress, associated PPE usage, and heat strain symptoms experienced when performing treatment and care activities [3]. The first part of the questionnaire gathered information about respondents’ demographic data, PPE usage, and heat exposure during the pandemic. The second part used a 5-point Likert scale (1 being “Strongly disagree” and 5 being “Strongly agree”) to investigate both HCWs’ knowledge about the effects of heat stress, and attitudes and practices towards PPE usage. The third part of the questionnaire examined heat strain symptoms experienced by HCWs while working in PPE.

### Content validity

Content validity was performed to assess the comprehensiveness of the items for measuring the constructs. Occupational and environmental medicine physicians and experts from epidemiology and statistics, environmental health, occupational health, and physiology were involved in this process. Ambiguous questions were rephrased for clarity. Local experts translated the English items into local languages while preserving their original meaning, fluency, and appropriateness.

### Construct validity

Construct validity for knowledge of heat stress, attitudes and practices regarding heat stress and PPE usage, and heat strain symptoms experienced was tested using exploratory factor analysis. Prior to factor extraction, Kaiser-Meyer-Olkin Measure of Sampling Adequacy and Bartlett’s Test of Sphericity were performed to assess the suitability of the data for factor analysis. Subsequently, factor extraction was performed using principal component analysis and varimax rotation. Factors with eigenvalues > 1 were extracted. Construct reliability was measured using Cronbach’s alpha. A Cronbach’s alpha ≥ 0.7 was considered to represent good internal consistency [13].

Confirmatory factor analysis was then conducted to verify the factorial structure of HCWs’ knowledge of heat stress, attitudes and practices regarding heat stress and PPE usage, and heat strain symptoms experienced. Analyses were performed using all items with factor loading > 0.5 [12]. The models’ goodness-of-fit was assessed using the following statistics: (i) Comparative Fit Index > 0.9, (ii) Goodness-of-Fit Index > 0.9, and (iii) Root-Mean-Square Error of Approximation < 0.08 [14].

### Convergent validity

Convergent validity was assessed by the average variance extracted, and the composite reliability of the questionnaire was calculated. Convergent validity was accepted if the average variance extracted > 0.5 and composite reliability > 0.7 [12, 15].

### Statistical analysis

Statistical analysis was performed using SPSS Statistics 18 (IBM Corp., Armonk City, NY, USA) to assess the construct validity and reliability. The “lavaan” package of R software was used for structural equation modelling [16], the “semTools” to calculate the average variance extracted value [17], and the “semPlot” package for drawing path diagram [18]. Continuous variables were expressed in mean ± standard deviation (SD) while categorical variables were expressed in percentages (%). A *p*-value less than 0.05 was considered to be statistically significant.

## Results

### Respondents’ demographic data and work conditions

The questionnaire was completed by 3,082 HCWs from six Southeast and South Asian countries and regions. Table 1 shows the breakdown of respondents’ demographic data, work conditions and PPE usage based on country or region. There were 2161 respondents from Taiwan, 110 from India, 407 from Indonesia, 55 from Singapore, 142 from Thailand, and 207 from Vietnam. The mean respondent age was 35.7 ± 10.3 years. 83.5% of the respondents were female. There were 339 medical doctors (11.0%), 2,630 nurses (85.3%), 16 technicians (0.5%), 22 sanitary workers (0.7%), and the remaining 75 workers held other roles (e.g., laboratory staff, radiologists) not previously mentioned (2.4%). Among respondents, 628 (20.4%) mainly worked in non-air-conditioned areas, and 215 respondents (7.0%) worked outdoors. There were 978 respondents (31.7%) that reported not having access to a dedicated rest area.

**Table 1:** Demographic information, work conditions, and use of personal protective equipment (PPE) among healthcare workers respondents

Characteristics	Taiwan (*n* = 2161)	India (*n* = 110)	Indonesia (*n* = 407)	Singapore (*n* = 55)	Thailand (*n* = 142)	Vietnam (*n* = 207)
Age (years)	37.9 ± 10.1	33.0 ± 9.8	32.5 ± 9.0	30.5 ± 9.0	33.8 ± 8.8	23.0 ± 2.0
Number of female participants	2076 (96.1%)	46 (41.8%)	290 (71.3%)	35 (63.6%)	106 (74.6%)	20 (9.7%)
Number of participants that worked mainly in a non-air-conditioned area	258 (11.9%)	83 (75.5%)	41 (10.1%)	21 (38.2%)	41 (28.9%)	184 (88.9%)
Number of participants required to work outdoors	172 (8.0%)	2 (1.8%)	0 (0.0%)	30 (54.5%)	0 (0.0%)	11 (5.3%)
Number of participants with a dedicated rest area	717 (33.2%)	67 (60.9%)	107 (26.3%)	0 (0.0%)	0 (0.0%)	87 (42.0%)
Use of PPE						
Number of participants that used an N95 or equivalent	1478 (68.4%)	93 (84.5%)	364 (89.4%)	55 (100.0%)	109 (76.8%)	166 (80.2%)
Number of participants that used a surgical mask	2018 (93.4%)	97 (88.2%)	380 (93.4%)	10 (18.2%)	61 (43.0%)	69 (33.3%)
Number of participants that used gloves	1995 (92.3%)	99 (90.0%)	383 (94.1%)	53 (96.4%)	102 (71.8%)	152 (73.4%)
Number of participants that used a gown	1871 (86.6%)	68 (61.8%)	368 (90.4%)	54 (98.2%)	108 (76.1%)	153 (73.9%)
Number of participants that used goggles	1413 (65.4%)	52 (47.3%)	260 (63.9%)	48 (87.3%)	22 (15.5%)	11 (5.3%)
Number of participants that used a face shield	1309 (60.6%)	38 (34.5%)	306 (75.2%)	14 (25.5%)	136 (95.8%)	145 (70.0%)
How many days in a week do HCWs work in PPE? (days)	3.5 ± 2.1	5.4 ± 1.5	5.1 ± 1.3	4.7 ± 1.0	5.3 ± 1.5	4.4 ± 3.4
How many hours do HCWs wear PPE for each shift? (h)	6.4 ± 3.5	6.4 ± 2.3	7.1 (3.3)	8.1 ± 1.0	5.3 ± 1.9	3.8 ± 6.6
Number of participants that take off PPE during breaks	335 (15.5%)	44 (40.0%)	202 (49.6%)	1 (1.8%)	0 (0.0%)	147 (71.0%)
Number of participants that took sick leave due to heat stress	79 (3.7%)	0 (0.0%)	19 (4.7%)	0 (0.0%)	0 (0.0%)	5 (2.4%)

Data expressed in n (%) for categorical variables, and mean ± standard deviation (SD) for continuous variables

### Use of personal protective equipment

Gloves were the most commonly used PPE (90.3%), followed by surgical masks (85.5%), gowns (85.1%), N95 or equivalent (73.5%), face shields (63.2%), and goggles (61.8%). The mean number of days in a week that respondents worked in PPE was 4.0 ± 2.2 days, and the mean number of hours that PPE was worn for each shift was 6.2 ± 3.3 h. 23.7% of respondents did not remove PPE during breaks.

### Knowledge about the effects of heat stress

**Fig. 1:**
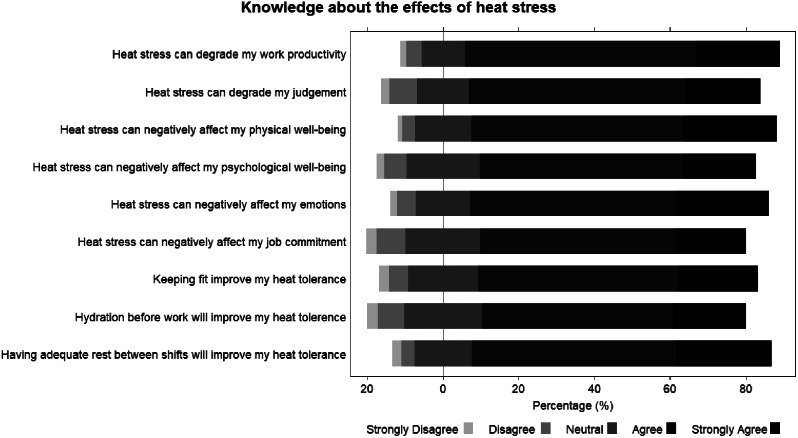
Healthcare workers’ responses to items on the knowledge about the effects of heat stress

Figure 1 shows the responses of the knowledge section of the questionnaire. For all items, more than 50% of the responses were “Agree” and “Strongly Agree”.

### Attitudes and practices regarding PPE usage

**Fig. 2:**
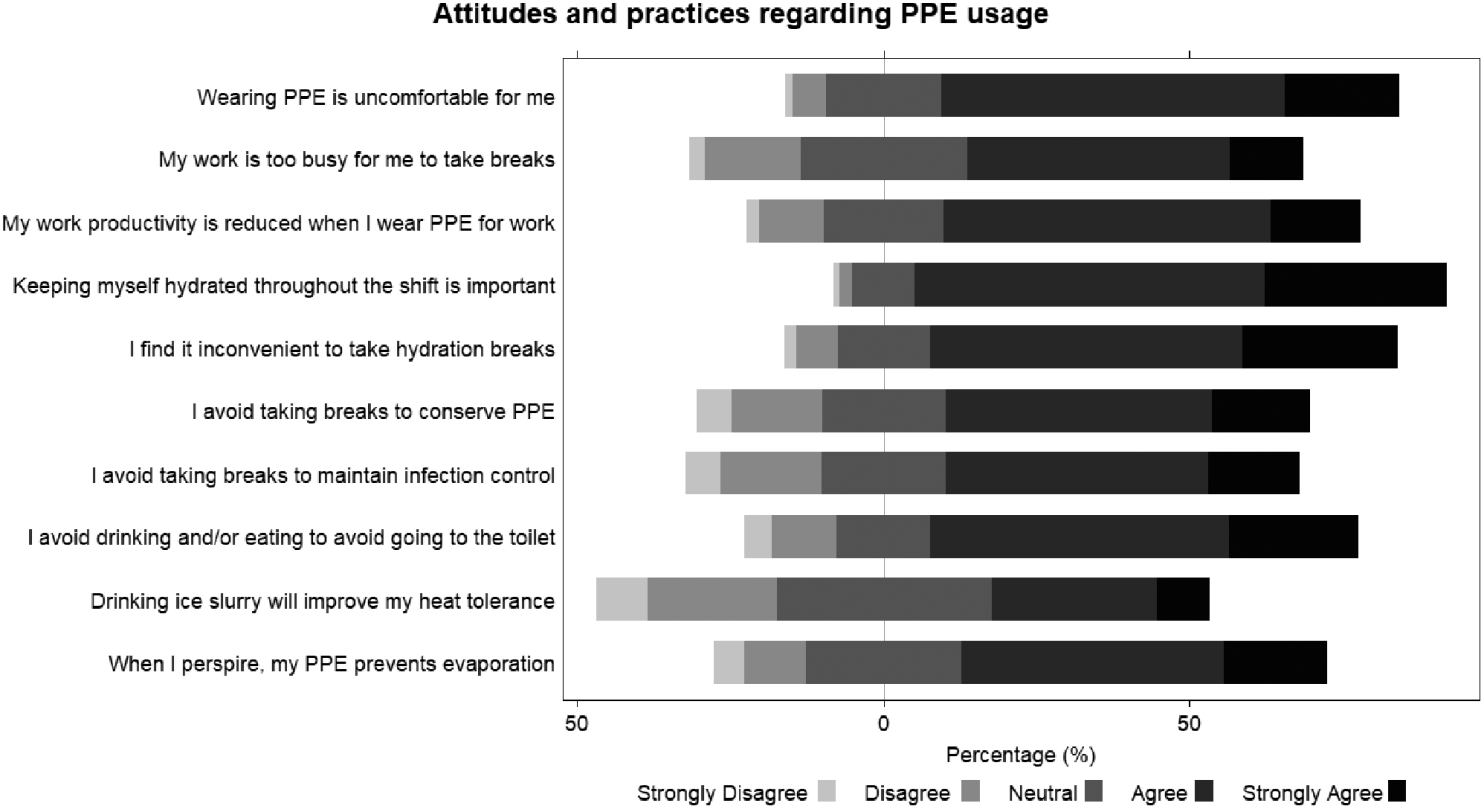
Healthcare workers’ responses to items on the attitudes and pratices regarding PPE usage

Figure 2 shows the responses of the attitudes and practices towards PPE usage section of the questionnaire. The responses “Agree” and “Strongly Agree” made up more than 50% of all responses for all items except for the statement “Drinking ice slurry will improve my heat tolerance” which majority responded “Neutral”.

### Heat strain symptoms

HCWs reported heat strain symptoms, including headache (28.2%), dizziness (37.2%), thirst (61.7%), vomiting (4.0%), excessive sweating (59.6%), breathing difficulty (31.8%), dehydration (21.1%), exhaustion (16.0%), and wanting to go to a more comfortable area (51.3%). Days spent wearing PPE per week correlated with the number of heat strain symptoms (*r* = 0.17, *p* < 0.001).

### Construct validity

Table 2 shows the results of exploratory factor analysis for knowledge (nine items) and attitudes and practices (seven items). The Kaiser-Meyer-Olkin Measure of Sampling Adequacy was 0.9, and the significant Bartlett’s value (*p* < 0.001) suggested that the data was suitable for factor analysis.

**Table 2:** Principal component analysis for the constructs knowledge, attitudes and practices, and heat strain symptoms, and their individual items

	Component and Factor Loading			Reliability Analysis		
	1	2	3	Item-Total Correlation	Cronbach's Alpha	
Constructs and Items					(Construct)	(Total)
Knowledge					0.9	0.9
k1. Heat stress can degrade my work productivity.	0.8			0.7		
k2. Heat stress can degrade my judgement.	0.8			0.6		
k3. Heat stress can negatively affect my physical well-being.	0.8			0.7		
k4. Heat stress can negatively affect my psychological well-being.	0.8			0.7		
k5. Heat stress can negatively affect my emotions.	0.8			0.7		
k6. Heat stress can negatively affect my job commitment.	0.8			0.6		
k7. Keeping fit improve my heat tolerance.	0.6			0.4		
k8. Hydration before work will improve my heat tolerance.	0.6			0.4		
k9. Having adequate rest between shifts will improve my heat tolerance.	0.6			0.4		
Attitudes and Practices					0.9	
a1. Wearing PPE is uncomfortable for me.		0.6		0.6		
a2. My work is too busy for me to take breaks.		0.6		0.6		
a3. My work productivity is reduced when I wear PPE for work.		0.6		0.6		
a4. Keeping myself hydrated throughout the shift is important.		0.2		0.4		
a5. I find it inconvenient to take hydration breaks.		0.7		0.6		
a6. I avoid taking breaks to conserve PPE.		0.8		0.5		
a7. I avoid taking breaks to maintain infection control.		0.8		0.5		
a8. I avoid drinking and/or eating to avoid going to the toilet.		0.8		0.5		
a9. Drinking ice slurry will improve my heat tolerance.		0.3		0.3		
a10. When I perspire, my PPE prevents evaporation.		0.4		0.5		
Heat strain symptoms					0.6	
s1. Headache			0.5	0.2		
s2. Dizziness			0.5	0.2		
s3. Thirst			0.5	0.2		
s4. Vomiting			0.4	0.1		
s5. Excessive sweating			0.5	0.1		
s6. Breathing difficulty			0.5	0.2		
s7. Dehydration			0.6	0.2		
s8. Exhaustion			0.5	0.1		
s9. Wanting to go to a more comfortable area			0.4	0.2		

### Reliability of questionnaire

Reliability analysis indicated Cronbach’s alpha of 0.9 for all items, 0.9 for knowledge, 0.9 for attitudes and practices, and 0.6 for heat strain symptoms, respectively (Table 2), which represents good internal consistency within the constructs of knowledge, and attitudes and practices, and poor internal consistency within the construct of heat strain symptoms.

### Convergent validity

Table 3 shows the convergent validity and composite reliability of the domains. Two of the three constructs were valid and reliable for measuring knowledge, and attitudes and practices towards PPE usage and heat stress.

**Table 3:** Item factor loading within each construct and the convergent validity and composite reliability of constructs

Construct	Item	Factor Loading	Average Variance Extracted	Composite Reliability
Knowledge	k1	0.6	0.5	0.9
	k2	0.7		
	k3	0.7		
	k4	0.8		
	k5	0.8		
	k6	0.8		
	k7	0.4		
	k8	0.3		
	k9	0.4		
Attitudes and Practices	a1	0.5	0.5	0.9
	a2	0.6		
	a3	0.6		
	a5	0.7		
	a6	0.9		
	a7	0.8		
	a8	0.8		
Heat strain symptoms	s1	0.2	0.2	0.5
	s2	0.3		
	s3	0.2		
	s5	0.1		
	s6	0.1		
	s7	0.2		
	s8	0.0		

**Fig. 3:**
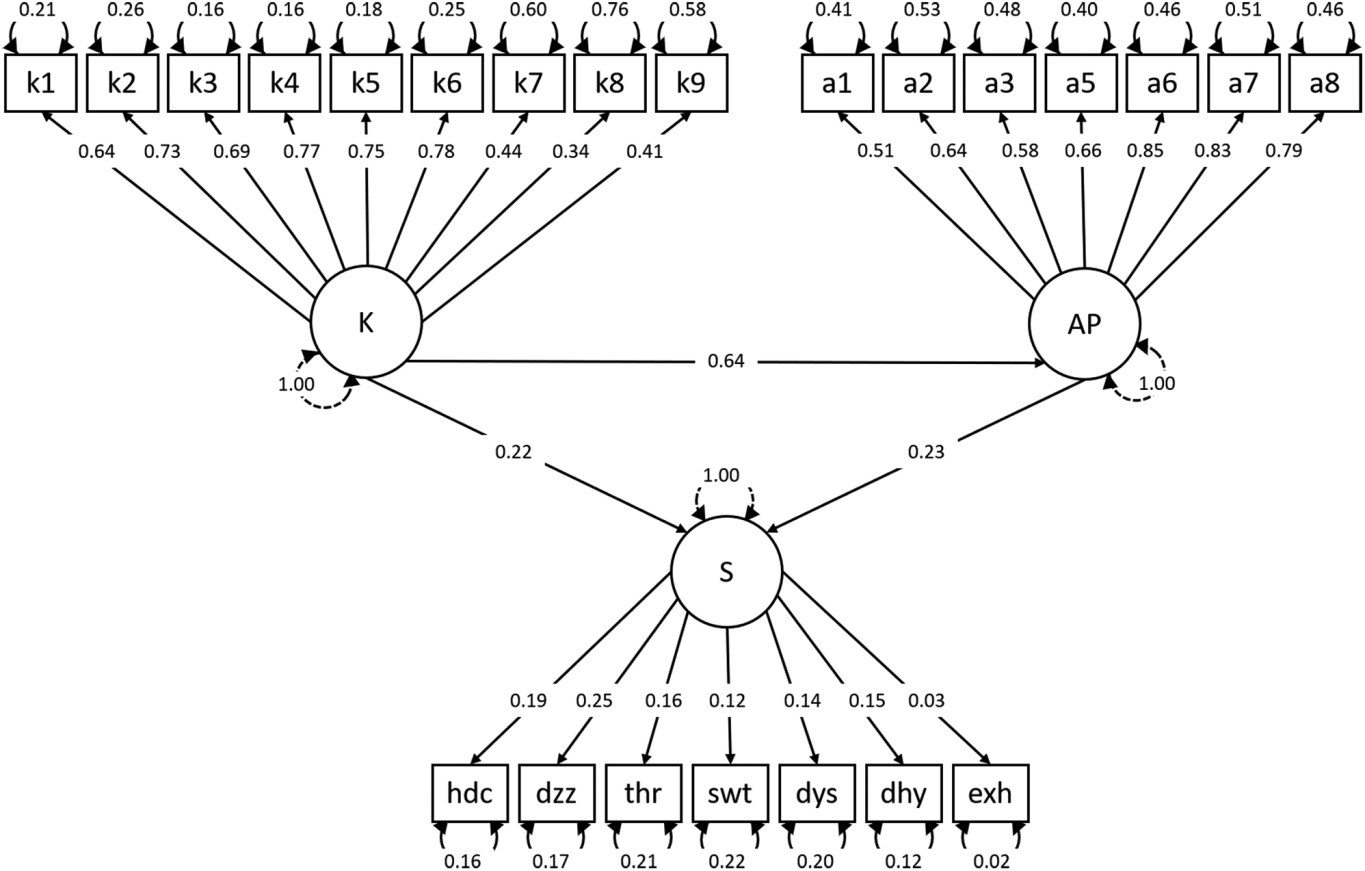
Confirmatory factor analysis of the knowledge, attitudes and practices, and heat strain symptoms model. Large circles represent latent variables and rectangles represent observed variables. Single arrows represent a uni-directional effect from one variable to another, while dual-head arrows represent factor covariances. Standardised path coefficients are indicated by the numbers above the arrows and represent the correlation or strength of the relationship between factors. Small circles represent variance in each observed variable that cannot be accounted for by the model, such as biological variability and measurement error. K: knowledge; AP: attitudes and practices; S: heat strain symptoms; hdc: headache; dzz: dizziness; thr: thirst; swt: excessive sweating; dys: breathing difficulty; dhy: dehydration; exh: exhaustion

**Fig. 4:**
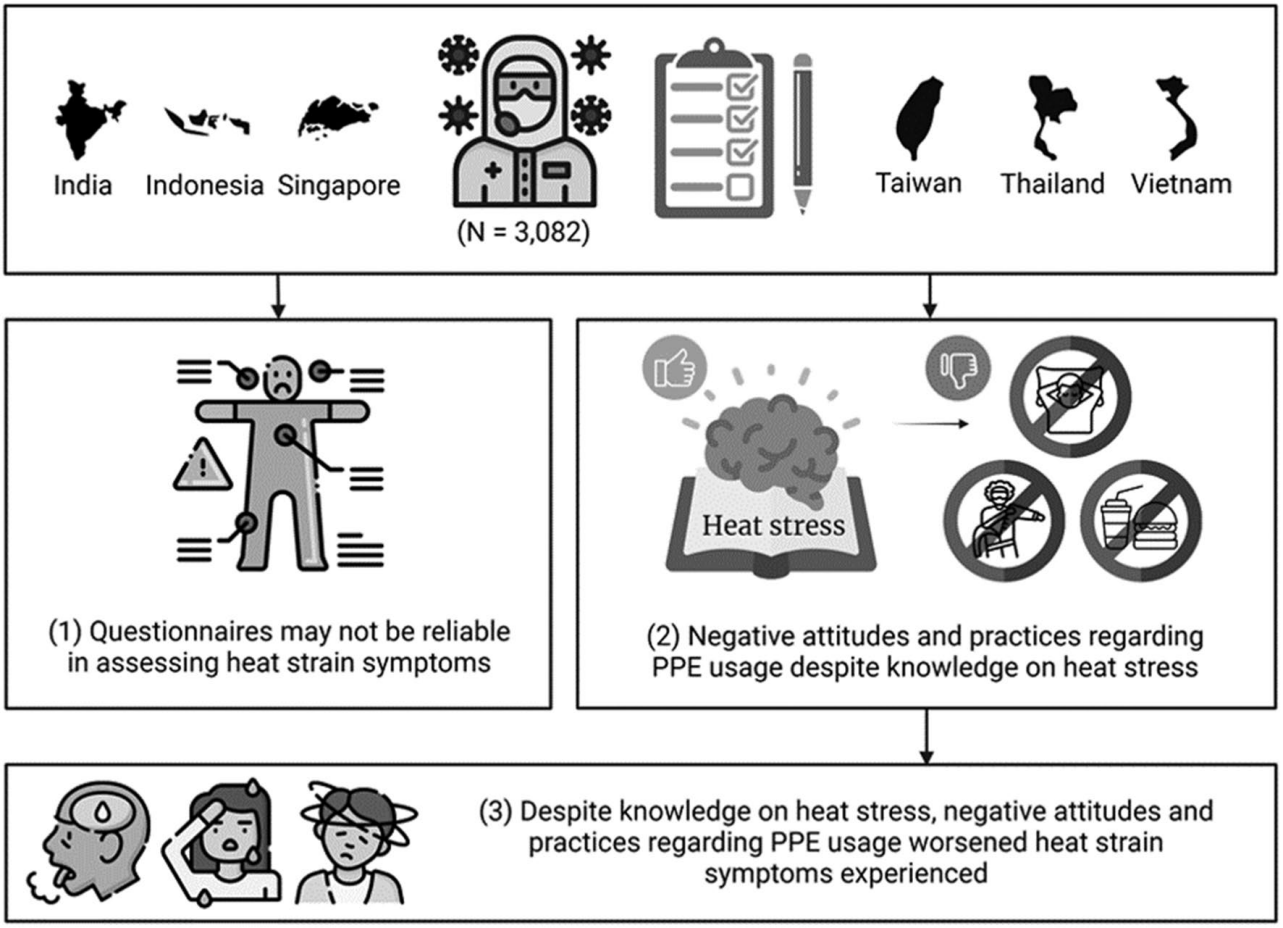
Summary of main findings from questionnaire administered to 3,082 healthcare workers

The structural model between the variables of knowledge, attitudes and practices, and heat strain symptoms is shown in Fig. 3; Table 3. The Comparative Fit Index was 0.8, Goodness-of-Fit Index was 0.8, and Root-Mean-Square Error of Approximation was 0.1, suggesting an acceptable model fit. There was a significant relationship between knowledge, and attitudes and practices (β1 = 0.6, *p* < 0.001) (Fig. 3). The magnitude and direction of the model suggested that knowledge (path coefficient = 0.2, *p* < 0.001), and attitudes and practices (path coefficient = 0.2, *p* < 0.001) directly affected heat strain symptoms (Fig. 3). “I avoid taking breaks to conserve PPE (a6)”, “I avoid taking breaks to maintain infection control (a7)” and “I avoid drinking and/or eating to avoid going to the toilet (a8)” were the most crucial factor contributing to heat strain symptoms (Table 2). Main findings are summarised in Fig. 4.

## Discussion

This study aimed to (i) to evaluate the heat stress perceived by HCWs engaged in different activities across Asia and (ii) to use factor analysis to validate a previously administered questionnaire on their perceptions of heat stress, associated PPE usage, and heat strain symptoms experienced. The authors found that HCWs displayed knowledge on the effects of heat stress, negative attitudes and practices regarding PPE usage, and experienced heat strain symptoms. From factor analysis, the questionnaire adequately and appropriately covered the constructs of knowledge, attitudes and practices, but not heat strain symptoms. There is also an association between knowledge of heat stress, and negative attitudes and practices towards PPE usage. Heat stress knowledge and negative attitudes towards PPE usage negatively affected the symptoms experienced by HCWs.

Based on the questionnaire results, HCWs displayed knowledge on the effects of heat stress. More than 72% of HCWs were aware that heat stress has potential negative physical, psychological, and emotional impacts. More than 65% of HCWs were also aware that heat stress can affect their work productivity, judgement, and commitment. Having knowledge on the effects of heat stress was also observed in other studies involving HCWs during the COVID-19 pandemic [3, 4, 19]. This should be expected of most HCWs involved in the questionnaire given that they are medical professionals. However, HCWs displayed negative attitudes and practices regarding PPE usage. More than half of HCWs responded that PPE usage caused discomfort and prevented sweat evaporation, and that they avoided taking breaks to conserve PPE and maintain infection control, and avoided drinking and/or eating to avoid going to the toilet. These negative attitudes and practices were also observed in other qualitative studies conducted during the pandemic [4, 20–22]. Poor attitudes and practices regarding PPE usage can increase HCWs’ heat stress exposure, especially when faced with a greater workload duringthe pandemic. This is exacerbated by rising global temperatures, especially if workers work outdoors, or do not have access to air-conditioning. From our study, more than three-quarters of HCWs in India and Vietnam were working in areas without air-conditioning. Taken together, the combination of increasing global temperatures and workload can increase the heat stress exposure faced by HCWs working in PPE, which can worsen their health and well-being during treatment and care activities.

The questionnaire was confirmed using factor analysis to be valid and reliable in measuring HCWs’ knowledge, attitudes and practices towards heat stress and PPE usage. Good internal consistency was observed within these two constructs. Good composite reliability and average variance extracted represented that the questionnaire had sufficient psychometric properties in the areas of knowledge, and attitudes and practices. This was not shown when the questionnaire was administered to HCWs in Singapore and India [3]. However, the questionnaire might not be reliable in assessing heat strain symptoms experienced by the HCWs. A possible reason is that symptoms were self-reported. As some respondents are not medical professionals, self-reported symptoms from these respondents might be inaccurate. Respondents might have also associated the symptoms experienced with other sources such as increased cardiovascular strain, and therefore under-reported heat strain symptoms experienced [3]. Additionally, the dichotomous nature of the items on heat strain symptoms might have caused underestimation of Cronbach’s alpha [23]. The self-reported and dichotomous nature of heat strain symptoms items can explain the low reliability of the questionnaire in assessing heat strain symptoms. Nonetheless, it is important to assess heat strain symptoms experienced by HCWs as early detection of heat strain can prevent the onset of serious heat illnesses. HCWs should be educated on the various heat strain symptoms, and encouraged to take rest or seek medical advice upon experiencing any of the symptoms when working in PPE. Using factor analysis to validate the current questionnaire allowed us to understand the interactions between knowledge, attitudes and practices, and symptoms in relation to heat stress and PPE usage, which could aid the development of better diagnostic tools and more specific heat management strategies.

Using factor analysis, an association was found between HCWs’ knowledge on heat stress and negative attitudes and practices regarding PPE usage. While HCWs have shown knowledge on heat stress [3], their negative attitudes and practices can be due to circumstances at work such as PPE shortages and long working hours during the pandemic [24]. In our study, almost one in four HCWs reported not removing PPE during breaks. Negative practices such as not taking breaks to conserve PPE or to maintain infection control, and not drinking and/or eating to avoid going to the toilet were most associated with heat strain symptoms. Adopting such negative practices can put HCWs at higher risk of heat illnesses. During a pilot study involving HCWs wearing PPE in a high-level isolation unit during a 4 h shift, half the participants had body core temperatures exceeding 38.5°C [25]. In particular, HCWs who avoid drinking and/or eating to avoid going to the toilet are at risk of dehydration and heat-induced kidney diseases [26]. Therefore, while HCWs understood the effects of heat stress, their negative practices towards PPE usage, along with poor attitudes displayed towards PPE usage, exposed them to occupational heat stress and could lead to negative health impacts.

From the structural model, the knowledge, and attitudes and practices displayed by HCWs on heat stress and its associated PPE usage negatively affected the symptoms experienced. Symptoms of thirst, excessive sweating, and dizziness were most experienced. These symptoms were also reported in other surveys administered to HCWs during the pandemic [4, 19, 27–30]. Similarly, the current study found that the number of heat strain symptoms increased with the number of days spent wearing PPE per week. Despite the inaccuracy of self-reported symptoms, it is essential for HCWs to acknowledge heat strain symptoms they experience. Compounded by the increasing COVID-19 cases requiring hospital care, HCWs who continue working despite thermal discomfort can negatively affect patients under their care due to poor decision-making. Therefore, targeted heat management strategies must be implemented to alleviate these heat strain symptoms.

In the various countries and regions where the questionnaire was administered, there were different safety regulations in place [3, 31, 32]. Based on questionnaire responses, some of the known adverse effects of PPE usage were already well understood by HCWs. However, the use of factor analysis revealed that knowledge on heat stress was not necessarily translated into actions due to the negative attitudes that HCWs displayed towards PPE. This suggests that employers should ascertain the underlying reasons behind HCWs’ negative attitudes and practices regarding PPE usage in order to enforce targeted heat management strategies.

Possible strategies to combat heat stress among HCWs that employers can consider include the provision of sufficient PPE, cool drinking water, and dedicated rest areas for donning and doffing PPE. To mitigate against dehydration and increased body core temperature due to occupational heat exposure [7, 33], cold water dispensers should be easily accessible to HCWs. In Singapore, pre-shift ingestion of ice slurry or cold water was suggested [3, 34], though this might depend on the context of application. Employers should also consider conducting training for donning and doffing of PPE, and managing HCWs’ expectations regarding PPE discomfort [35], which may improve attitudes and practices regarding PPE usage. With global temperatures expected to rise further, implementation of targeted management strategies, and availability of adequate resources, are necessary during a pandemic to protect healthcare workers from heat exposure due to PPE usage and their added workload. Ensuring the health and well-being of HCWs can facilitate a better healthcare system for patient care, especially in the face of global warming and in the event of future pandemics.

### Limitations

The authors would like to acknowledge some limitations in this study. Firstly, there might be non-response bias as the survey was conducted voluntarily. Secondly, self-reported data was collected in this study, which relied on the respondents’ memory and could be subjected to social desirability bias, resulting in HCWs over-reporting good practices despite not actually adhering to them. Thirdly, the symptoms HCWs experienced might not have been fully understood due to the low reliability in the symptoms construct. This might have resulted in under-reporting or over-reporting of heat stress symptoms experienced. Future research should aim to develop a better assessment tool for understanding such symptoms.

## Conclusion

In summary, self-reported symptoms might not be as accurate as diagnostic assessments, However, HCWs should still self-assess their symptoms for early management of heat stress. Despite knowledge on heat stress, it was suggested that negative attitudes and practices regarding PPE usage contributed to the heat strain symptoms HCWs experienced. Further empirical research is still warranted to ascertain any causative effects. To further improve the understanding of PPE usage and its associated heat stress, healthcare institutions should continue to educate HCWs on occupational heat stress. Additionally, understanding that HCWs’ negative attitudes and practices can lead to occupational heat stress exposure is essential when implementating targeted health management policies and to ensure adequate resources are available for HCWs to manage heat stress. This uncovers an important lesson learnt during the current pandemic, which is relevant and applicable for future emergencies. Further research should aim to investigate the main issues in each country or region in order for more targeted solutions to be implemented to ensure that HCWs caring for patients are also being cared for.

### Electronic supplementary material

Below is the link to the electronic supplementary material.

Supplementary Material 1

## Data Availability

The datasets used and/or analysed during the current study are available from the corresponding author on reasonable request.

## List of Abbreviations

COVID-19: Coronavirus disease 2019
HCW: Healthcare worker
PPE: Personal protective equipment
