# Supplementary material for: Poor personal protective equipment practices were associated with heat-related symptoms among Asian healthcare workers: a large-scale multi-national questionnaire survey
Source: BMC Nurs. 2024 Mar 1;23:145. doi: 10.1186/s12912-024-01770-x (PMC10905833; doi:10.1186/s12912-024-01770-x)
Supplement: Supplementary file 1 — Supplementary Material 1 [file 12912_2024_1770_MOESM1_ESM.pdf]

Personal Protective Equipment (PPE) and Thermal Stress Questionnaire

* Required

1. Date *

____

Example: January 7, 2019

2. City *

____

Demographic Details

3. Age (in years) *

____

4. Sex *

Mark only one oval.

- ( ) Male
- ( ) Female

5. Weight (in kilograms) *

____

6. Height (in metres) *

____

7. Role in the organisation *

Mark only one oval.

- Medical
- Nursing
- Operations
- Sanitary worker
- Other: ____________________

8. Location of work *

Mark only one oval.

- Tentage
- Fever Facility
- Clean Area
- Other: ____________________

9. Are you fasting for Ramadan or other religious events? *

Mark only one oval.

- Yes
- No

PPE Use and Heat Exposure

10. Which type of PPE do you use at work? *

Check all that apply.

- [ ] N95 or equivalent
- [ ] Surgical mask
- [ ] Gloves
- [ ] Gown
- [ ] Goggles
- [ ] Face shield

Other: [ ] ______________________

11. How many days in a week do you work in PPE? *

____

12. How many hours do you wear PPE for each shift? *

____

13. Do you mainly work in an air conditioned or non-air conditioned area? *

Mark only one oval.

- ( ) Air conditioned
- ( ) Non-air conditioned

14. How long does it take to put on your PPE? (in minutes) *

____

15. Have you had to take sick leave due to heat stress? *

Mark only one oval.

- ( ) Yes
- ( ) No

16. If you answered 'Yes' for the previous question, for how many days of sick leave have you taken in the last 3 months?

____

Behaviour and Adaptation to Thermal Stress

17. Do you take your PPE off during breaks? *

Mark only one oval.

- ( ) Yes
- ( ) No

18. Do you have a dedicated rest area? *

Mark only one oval.

- ( ) Yes
- ( ) No

19. What would be your best way to reduce heat stress when you are using PPE?

____

20. Which symptoms do you experience while working in PPE? *

Check all that apply.

- [ ] Headache
- [ ] Dizziness
- [ ] Thirst
- [ ] Vomitting
- [ ] Excessive sweating
- [ ] Breathing difficulty
- [ ] Dehydration
- [ ] Exhaustion
- [ ] Wanting to go a more comfortable area

Other: [ ] ______________________

Knowledge about Effects of Thermal Stress

21. Heat stress can degrade my work productivity *

Mark only one oval.

	1	2	3	4	5	
Strongly Disagree	○	○	○	○	○	Strongly Agree

22. Heat stress can degrade my judgement *

Mark only one oval.

	1	2	3	4	5	
Strongly Disagree	○	○	○	○	○	Strongly Agree

23. Heat stress can negatively affect my physical well-being *

Mark only one oval.

	1	2	3	4	5	
Strongly Disagree	⬭	⬭	⬭	⬭	⬭	Strongly Agree

24. Heat stress can negatively affect my psychological well-being *

Mark only one oval.

	1	2	3	4	5	
Strongly Disagree	⬭	⬭	⬭	⬭	⬭	Strongly Agree

25. Heat stress can negatively affect my emotions *

Mark only one oval.

	1	2	3	4	5	
Strongly Disagree	⬭	⬭	⬭	⬭	⬭	Strongly Agree

26. Heat stress can negatively affect my job commitment *

Mark only one oval.

	1	2	3	4	5	
Strongly Disagree	⬭	⬭	⬭	⬭	⬭	Strongly Agree

27. Keeping fit will improve my heat tolerance *

Mark only one oval.

	1	2	3	4	5	
Strongly Disagree						Strongly Agree

28. Hydration before work will improve my heat tolerance *

Mark only one oval.

	1	2	3	4	5	
Strongly Disagree						Strongly Agree

29. Having adequate rest between shifts will improve my heat tolerance *

Mark only one oval.

	1	2	3	4	5	
Strongly Disagree						Strongly Agree

Attitude towards PPE Use during Pandemic and its Thermal Stress Effects

30. Wearing PPE is uncomfortable for me *

Mark only one oval.

	1	2	3	4	5	
Strongly Disagree						Strongly Agree

31. My work is too busy for me to take breaks *

Mark only one oval.

	1	2	3	4	5	
Strongly Disagree	⬭	⬭	⬭	⬭	⬭	Strongly Agree

32. My work productivity is reduced when I wear PPE for work *

Mark only one oval.

	1	2	3	4	5	
Strongly Disagree	⬭	⬭	⬭	⬭	⬭	Strongly Agree

33. Keeping myself hydrated throughout the shift is important *

Mark only one oval.

	1	2	3	4	5	
Strongly Disagree	⬭	⬭	⬭	⬭	⬭	Strongly Agree

34. I find it inconvenient to take hydration breaks *

Mark only one oval.

	1	2	3	4	5	
Strongly Disagree	⬭	⬭	⬭	⬭	⬭	Strongly Agree

35. I avoid taking breaks to conserve PPE *

Mark only one oval.

	1	2	3	4	5	
Strongly Disagree	⬭	⬭	⬭	⬭	⬭	Strongly Agree

36. I avoid taking breaks to maintain infection control *

Mark only one oval.

	1	2	3	4	5	
Strongly Disagree	⬭	⬭	⬭	⬭	⬭	Strongly Agree

37. I avoid drinking and/or eating to avoid going to the toilet *

Mark only one oval.

	1	2	3	4	5	
Strongly Disagree	⬭	⬭	⬭	⬭	⬭	Strongly Agree

38. Drinking ice slurry will improve my heat tolerance *

Mark only one oval.

	1	2	3	4	5	
Strongly Disagree	⬭	⬭	⬭	⬭	⬭	Strongly Agree

39. When I perspire, my PPE prevents sweat evaporation *

Mark only one oval.

	1	2	3	4	5	
Strongly Disagree	⬭	⬭	⬭	⬭	⬭	Strongly Agree
